# Correlations of oral behaviors, pain, and psychological distress among patients with temporomandibular disorders: clinical investigation of axial II evaluation

**DOI:** 10.3389/fpubh.2025.1604995

**Published:** 2025-06-24

**Authors:** Yan Yu, Lili Wang, Chunxia Chen, Mengmeng Wang, Shuai Nie, Lin Fu

**Affiliations:** ^1^Department of Prosthodontics, Tianjin Stomatological Hospital, School of Medicine, Nankai University, Tianjin, China; ^2^Tianjin Key Laboratory of Oral and Maxillofacial Function Reconstruction, Tianjin, China; ^3^Department of Endodontics, Tianjin Stomatological Hospital, School of Medicine, Nankai University, Tianjin, China; ^4^School of Sociology, Beijing University of Technology, Beijing, China; ^5^Beijing Social Governance Research Center, Beijing University of Technology, Beijing, China

**Keywords:** temporomandibular disorders, psychological distress, pain, oral behavior, axial II

## Abstract

**Purpose:**

This cross-sectional, observational study assessed physical, psychological and behavioral factors related to patients with temporomandibular disorder (TMD) using Axis II assessment instruments with diagnostic criteria for TMD (DC/TMD) and investigated the correlations among oral behavior, pain, and psychological distress in patients with temporomandibular disorders.

**Methods:**

Participants were recruited from the Department of TMD at the authors’ hospital. The TMD group comprised 96 patients (27 males and 69 females; mean age 39.10 ± 10.83 years), stratified into three clinical subgroups based on primary symptoms: myofascial pain dysfunction syndrome (MPDS; subgroup 1), internal derangement (subgroup 2), and osteoarthritis (subgroup 3). The control group consisted of 111 individuals (42 males and 69 females; mean age 35.05 ± 7.94 years) with no history of TMD. Demographic information, oral behaviors, pain, and psychological outcomes of the TMD group were assessed using self-report questionnaires according to the diagnostic criteria of axial II. The non-TMD group was evaluated using similar instruments except for pain and jaw functional limitations.

**Results:**

Compared with the non-TMD group, the TMD group presented significantly greater levels of unhealthy oral behaviors and psychological outcomes, including anxiety, depression, and somatic symptoms. Within the TMD group, participants diagnosed with MPDS were found to have more psychological symptoms than those diagnosed with internal derangement and osteoarthritis. The psychological outcomes of patients with TMD were shown to be significantly positively correlated with their pain status and oral behaviors (*rs* = 0.23 ~ 0.52). The indirect effect of chronic pain was found to be significant in the relationships between oral behaviors and psychological outcomes, including anxiety, depression, and somatic symptoms.

**Conclusion:**

Oral behaviors and chronic pain were closely associated with psychosocial distress among patients with TMD. The effect of oral behavior on psychological distress is exerted indirectly through chronic pain. This study contributes to the conceptual framework for the development of individualized diagnostic and therapeutic strategies for patients with TMD.

## 1 Introduction

Temporomandibular disorders (TMDs) are a cluster of clinical problems involving the masticatory muscles, the temporomandibular joint (TMJ) and associated structures (1). Previous epidemiological surveys have shown that TMD signs/symptoms are present in 33 to 75% of the general population (2, 3). The prevalence of TMD in China is similar, ranging from 28 to 88%, with the majority of patients aged 20–30 years old and mostly being female (4, 5). The symptoms of TMD include orofacial and periauricular pain, joint clicking, and limitations in jaw movement and function. These symptoms can make patients’ eating or facial expressions difficult and can have negative effects on health, well-being, and quality of life. According to the main symptoms and sources of pain, TMDs can be divided into myofascial pain dysfunction syndrome (MDPS), internal derangement, and osteoarthritis. Different subtypes are distinguished in terms of diagnosis and treatment (6), all of which have adverse effects on patients.

The International Association for Dental Research developed the Diagnostic Criteria for TMD (DC/TMD) based on symptom questionnaires and clinical examinations, employing a biopsychosocial model for the conceptualization and diagnosis of TMDs (2). The clinical classification and diagnostic criteria of TMD consists of two diagnostic algorithms: Axis I encompass the standardized clinical procedures used to establish the clinical diagnosis of temporomandibular joint disorders while Axis II concerns self-report questionnaires assessing especially the psychological impact of TMD. The Axis I algorithms remain predominant in TMD diagnosis (7, 8), while Axis II algorithms provide physicians with a standardized method to assess psychological and behavioral factors (including chronic pain characteristics, oral parafunctional behaviors, jaw functional limitations, and psychiatric comorbidities) (2), enabling identification of risk factors (9, 10) and complement Axis I’s clinical diagnosis. The importance of Axis II evaluation for psychological and behavioral status has been increasingly recognized, as empirical studies have demonstrated that TMD patients tend to show more severe mental symptoms (11–14) and oral parafunction (15, 16).

Although current evidence cannot definitively establish a causal relationship between temporomandibular disorders (TMD) and psychological comorbidities, incorporating psychological assessment remains clinically crucial for TMD diagnosis and management. Studies report that 60–77% of TMD patients experience moderate-to-severe psychological distress (e.g., depression, somatization), which is associated with poorer treatment adherence, higher healthcare utilization, and significantly reduced quality of life independent of pain severity (11). This further substantiates that precise TMD diagnosis necessitates a multidimensional evaluation incorporating both behavioral and psychological dimensions to elucidate potential risk factors (9, 10). Furthermore, the prevalent underreporting of psychological symptoms among TMD patients poses significant diagnostic challenges for clinicians (17). This “hidden burden” often results in delayed interventions, and may increase long-term disability risks and socioeconomic costs (18). A prospective cohort study of 3,000 healthy (non-TMD) subjects demonstrated that depressive symptoms significantly increased the risk of temporomandibular joint pain, while anxiety symptoms were strongly associated with both joint and muscle pain (19). These findings underscore the importance of establishing clear diagnostic criteria for TMD and implementing them in clinical practice.

Although the pathogenesis of TMD is multifacial and unclear, oral behaviors are still considered among the factors that contribute to the onset and persistence of painful TMDs (20). Oral behaviors, involving multiple activities related to the mouth and jaws, have been found to be associated with psychological outcomes, including anxiety and depression (21–23). A prior study also demonstrated these connections by examining the correlations of sleeping/waking-state oral activities with different psychological factors (15). Another study of TMD subgroups revealed near-moderate correlations between overall/waking-state nonfunctional oral behaviors and depression/anxiety across different TMD subtypes among patients (24). While these studies consistently demonstrate associations between oral behaviors and psychological distress in TMD, the underlying mechanisms - particularly the potential indirect pathways - remain to be elucidated.

Pain, as one of the primary symptoms of TMD, may function as a critical mediator between oral behaviors and psychological distress. In clinical practice, pain constitutes the primary treatment motivator (18), owing to its multifaceted impacts on daily functioning, psychosocial well-being, sleep quality, and overall life satisfaction (2, 11, 25–27). Existing literature suggests two distinct pathways: The first involves oral parafunctional behaviors are established risk factors for TMD-related pain development (28–32), whereas the second implicates chronic orofacial pain may directly contribute to the development and exacerbation of psychological distress through sustained nociceptive input and functional impairment (18, 33–36). This postulated pathway finds indirect support in clinical observations that pain-focused interventions, including occlusal splint therapy and analgesic medications, effectively reduce pain and thereby enhance psychological outcomes (37).

These clinical observations lead us to preliminarily explore the hypothesized mechanism whereby oral behaviors may influence psychological distress through pain pathways. Using a cross-sectional design, this study aims to: (1) identify differences in Axis II diagnostic algorithm indicators between individuals with and without TMD; (2) examine correlations among jaw function, oral behaviors, pain, and psychological symptoms in TMD patients; and (3) explore the potential mediating role of pain. The study hypothesized that pain would exhibit an indirect effect in the relationship between oral behaviors and psychological distress among TMD patients, providing evidence to inform personalized clinical diagnosis and treatment approaches.

## 2 Materials and methods

### 2.1 Study population

Patients who first visited the temporomandibular joint (TMJ) clinic at the dental hospital where the author worked from June 2022 to April 2023 were selected. Patients seeking TMD treatment who had the following clinical symptoms were invited to participate in the survey: (1) joint clicking; (2) pain in the temporomandibular joint area or masticatory muscle; (3) abnormal jaw movement; and (4) any other symptoms related to TMD. All patients were diagnosed through clinical examination (according to Axis I), CBCT or joint MRI, divided into three clinical subgroups including myofascial pain dysfunction syndrome (MPDS; subgroup 1), internal derangement (subgroup 2), and osteoarthritis (subgroup 3). The inclusion criteria were as follows: (1) patients diagnosed with TMD by clinical and X-ray examination; (2) patients aged 18 years or above; and (3) patients able to understand and complete the questionnaire independently. The exclusion criteria were as follows: (1) patients with TMJ infection, tumor, or trauma; (2) patients with systemic arthritis; (3) patients with odontogenic pain or pain caused by nerve damage; (4) patients who were pregnant or had severe systemic diseases; and (5) patients with psychiatric disorders. The sample size of TMD patients was determined via Monte Carlo power analysis for the indirect effect (38). Based on prior evidence of pairwise correlations among variables including oral behaviors, pain, and psychological outcomes (*r* ≈ 0.3–0.6) (13, 15, 24), simulations indicated that a sample size of 93 achieved a power of ≥ 0.80 (when *n* = 93, *Power* = 0.82, *[LL, UL]* = [0.80, 0.85]) to detect the mediated effect. To ensure robustness, the researchers targeted a sample of 93–120 participants, ultimately recruiting 96 into the experimental group.

In addition, subjects who were free of TMD and whose medical history did not reveal any orofacial discomfort composed the control group. Recruitment for the control group involved employees and patients awaiting treatment of prosthodontic from the Stomatological Hospital in which the authors worked. Patients with psychiatric disorders were also excluded from the control group.

A total of 243 people were recruited (TMD group: *n* = 126; non-TMD group: *n* = 117). Among the TMD group, 30 patients were excluded due to incomplete questionnaires (*n* = 25), meeting exclusion criteria (*n* = 5). Six patients were excluded from the control group because they did not complete the survey.

### 2.2 Study procedures

This study was approved by the ethics committee of author’s institution (PH2022-B-010). Data collection was conducted by a trained research assistant using standardized protocols. Participant recruitment occurred in the TMJ clinic waiting area. First-visit outpatients awaiting physician consultation received a study information sheet and an introduction about the questionnaires, including the Jaw functional limitation scale (JFLS), Oral Behaviors Checklist (OBC), The Graded Chronic Pain Scale (GCP), General Anxiety Disorder Scale-7 (GAD-7), Patient Health Questionnaire-9 (PHQ-9) and Patient Health Questionnaire-15 (PHQ-15). After providing written informed consent, participants completed questionnaires with the assistant available to clarify questions. All participants were assured of data confidentiality, voluntary participation with the right to withdraw without penalty, and exclusive use of data for research purposes.

Participants in the control group were recruited through email and online advertisements. Specifically, researchers distributed recruitment emails to all employee mailboxes within the institution; additionally, patients awaiting treatment of prosthodontic who confirmed having no history of TMD diagnosis or any related symptoms were also eligible to participate. Eligible individuals who self-reported meeting the inclusion criteria provided electronic informed consent and completed the online questionnaires. As control group participants had no TMD-related pain or mandibular functional limitations, they were exempted from completing the JFLS and GCP scales.

### 2.3 Measures

#### 2.3.1 Jaw functional limitation scale

Jaw function was assessed by the Jaw Functional Limitation Scale (JFLS), a 20-item self-report scale that measures jaw function over the previous 30 days (39). The JFLS indicates overall jaw function limitations (biting something hard or soft, yawning, opening wide, making faces, etc.) and consists of subscales for three types of functional limitations, including *mastication* (six items), *vertical jaw mobility* (four items) and *verbal/nonverbal communication* (eight items). The items were rated from 0 (no limitations) to 10 (extreme limitations), with higher scores indicating greater degrees of jaw function disability. The JFLS has been validated and utilized extensively in the general population for the assessment of jaw functional limitations (40).

#### 2.3.2 Oral behaviors

Oral behaviors were measured using the Oral Behaviors Checklist (OBC), a self-report scale that assesses the frequency of oral behaviors performed during the preceding month. The OBC consists of 21 items and evaluates the frequency of sleeping-state and waking-state oral behaviors, such as tooth grinding, clenching, and gum chewing. Participants responded to the items on a five-point Likert-type scale ranging from 0 (*none of the time*) to 4 (*all the time*), with higher mean scores indicating a higher level of self-assessed unhealthy oral behavior. The instrument is considered reliable and valid for evaluating oral behaviors among Chinese individuals (41).

#### 2.3.3 Pain status

The Graded Chronic Pain Scale (GCPS) was used to assess pain status. It is mainly used to evaluate the facial pain status of TMD patients in the past 1–6 months. Version 2.0 of the GCPS (6 months) was published in 2011 (42). In addition to the 3 items for pain intensity and 4 items for function, one item was added to measure the number of days of pain. The scale consists of 8 items, each item is scored from 0 to 10, with 0 indicating no effect and 10 indicating inability to perform any activity.

Characteristic Pain Intensity (CPI): compute mean of items 2–4 (pain right now, worst pain, average pain), and multiply by 10, where 0 is no pain, 1–50 is low pain, and 51–100 is high pain.

Total Disability Points (TDP): the score of Points for Disability Days plus the score of Points for Interference.

Chronic Pain Grade (GCP): It was based on the CPI and TDP score, and divided into grades 0, I, II, III and IV. The CPI score of 0 was class 0; the CPI score of < 50 points and TDP score of < 3 points was class I; the CPI score of ≥ 50 points and TDP score of < 3 points was class II; the TDP score of 3–4 points was class III; and TDP score of 5–6 points was class IV.

#### 2.3.4 Anxiety symptoms

The anxiety symptoms of the participants were measured via the Generalized Anxiety Disorder Scale (GAD-7), a self-reported scale used to screen for anxiety disorders. The GAD-7 comprises 7 items designed to be used in general practice with accurate results. Each item was rated on a 4-point Likert scale ranging from 0 (*not at all*) to 3 (*nearly every day*), with higher scores indicating greater severity of anxiety.

#### 2.3.5 Depressive symptoms

Depressive symptoms in the past 2 weeks were measured via the nine-item Patient Health Questionnaire (PHQ-9), which is a self-reported scale consisting of 9 items. Each item is scored from 0 to 3 points, for a total possible score of 27 points. Scores of 0 ~ 4 points indicate no depression, scores of 5 ~ 9 points indicate mild depression, scores of 10 ~ 14 points indicate moderate depression, scores of 15 ~ 19 points indicate possible moderate to severe depression, and scores of 20 ~ 27 points indicate possible moderate to severe depression.

#### 2.3.6 Somatization symptoms

The Patient Health Questionnaire-15 (PHQ-15) is used to evaluate the presence of somatization symptoms. A total of 15 items are included, each of which is scored 0–2 points for a total score of 30 points. Nonspecific physical symptoms in the past 4 weeks were assessed as the degree of distress, with a total score of 0–4 points. A total score of 0–4 was considered normal, a score of 5–9 was considered mild, a score of 10–14 was considered moderate, and a score of 15–30 was considered severe.

#### 2.3.7 Demographic data

The demographic variables, including sex (*1 = male, 2 = female*), marital status *(1 = unmarried, 2 = married, 3 = other)*, education level *(1 = high school or below, 2 = junior college, 3 = college, 4 = postgraduate or above)*, occupation (*1 = student, 2 = civil servant, 3 = teacher, 4 = officer, 5 = worker, 6 = retired, 7 = other*) and age, were obtained through self-report questionnaires.

### 2.4 Statistical analyses

SPSS statistical software 26.0 (IBM Corporation, Armonk, New York, United States) was used for all the statistical analyses, with the significance level set at 0.05. Data are reported as frequencies with percentages and means/medians with standard deviations (SDs). The Pearson correlation coefficient was used for correlation analysis.

Prior to exanimating the hypothesized mediation model, we evaluated demographic variables should be controlled as covariates. The mediation analysis followed Hayes’ procedure (43). First, multiple regression analyses were conducted to assess direct and indirect effects. After controlling for covariates, oral behavior was included as a predictor in the regression model to examine its association with psychological distress (Model 1, 3, 5. Subsequently, pain was added stepwise as an additional predictor to test its effects while controlling for both covariates and oral behavior (Model 2, 4, 6). To further validate the mediation effects, bootstrapping analyses (5,000 resamples) were performed using PROCESS v4.0 (44), quantifying direct, indirect, and total effects (direct + indirect). Effect sizes were calculated using completely standardized coefficients. The significance of indirect effects was determined by examining 95% bias-corrected bootstrap confidence intervals; effects were considered statistically significant (*p* < 0.05) if the 95% confidence interval excluded zero. Bootstrapping mediation analyses were conducted in the current study instead of structural equation modeling (SEM) because bootstrapped confidence intervals in PROCESS demonstrate lower bias when estimating indirect effects in small samples compared to SEM (45).

## 3 Results

### 3.1 Demographic data

The demographic data of the final sample of 207 participants (138 females) are shown in Table 1. The “with TMDs” (TMD) group consisted of 96 subjects (27 males; 69 females) aged 39.1 ± 10.83 years. The “no TMDs” (NT) group comprised 111 subjects (42 males; 69 females) with a mean age of 35.05 ± 7.94 years. There was no significant difference between the TMD and non-TMD groups in terms of sex, marital status, education level, or occupation, while participants in the TMD group were generally older than those in the non-TMD group.

**Table 1:** Descriptive characteristics of the participants (*n* = 207).

Demography	TMD group (*n* = 96)	non-TMD group (*n* = 111)	t/χ^2^	*p*-value
Sex			2.185	0.139
Male	27 (28.1)	42 (37.8)		
Female	69 (71.9)	69 (62.2)		
Age	39.1 (10.83)	35.05 (7.94)	**3.101**	**0.002**
Marital status			−1.371	0.172
Unmarried	14 (14.6)	33 (29.7)		
Married	75 (78.1)	72 (64.9)		
Other	7 (7.3)	6 (5.4)		
Occupation			−0.805	0.421
Student	24 (25.0)	32 (28.8)		
Civil servant	22 (22.9)	11 (9.9)		
Teacher	11 (11.5)	13 (11.7)		
Officer	8 (8.3)	11 (9.9)		
Worker	21 (21.9)	32 (28.8)		
Retired	3 (3.1)	3 (2.7)		
Other	7 (7.3)	9 (8.1)		
Education level			1.56	0.120
High school or below	3 (3.1)	7 (6.3)		
Junior college	27 (28.1)	40 (36.0)		
Collage	54 (56.3)	52 (46.8)		
Postgraduate or above	12 (12.5)	12 (10.8)		

Bold values indicate statistically significant results.

### 3.2 Descriptive information and differences in outcomes between the TMD and non-TMD groups

The descriptive analyses of the two groups are depicted in Table 2, including the scores of oral behaviors, anxiety, depression and somatic symptoms of the two groups and the jaw function and pain status of the TMD groups. Significant differences in oral behaviors (*t* = 6.67, *p* < 0.001), somatization (*t* = 7.81, *p* < 0.001), depression (*t* = 8.73, *p* < 0.001) and anxiety (*t* = 11.70, *p* < 0.001) were detected between the non-TMD and TMD groups. Compared with the non-TMD group, the TMD group presented considerably greater levels of psychological distress.

**Table 2:** Difference tests for outcomes between the TMD and non-TMD groups.

Variables	Total (*n* = 207)	TMD (*n* = 96)	non-TMD (*n* = 111)	*t*	*p*-value
	*M ± SD*	*M ± SD*	*M ± SD*		
OBC	17.4 ± 7.87	20.96 ± 6.10	14.32 ± 7.95	6.67	**< 0.001**
PHQ-9	7.16 ± 4.96	9.93 ± 4.03	4.77 ± 4.42	8.73	**< 0.001**
GAD-7	5.38 ± 4.21	8.16 ± 3.27	2.97 ± 3.38	11.17	**< 0.001**
PHQ-15	7.6 ± 5.17	10.26 ± 4.34	5.31 ± 4.73	7.81	**< 0.001**

OBC, Oral Behaviors Checklist; PHQ-9, Patient Health Questionnaire-9; GAD-7, Generalized Anxiety Disorder Scale; PHQ-15, Patient Health Questionnaire-15. Bold values indicate statistically significant results.

Difference analyses of the three TMD subgroups revealed no significant differences in oral behavior, jaw function status, or chronic pain among TMD subgroups. However, patients in the MPDS subgroup demonstrated significantly higher scores on psychological symptoms including PHQ-9 (*F* = 5.97, *p* = 0.004), GAD-7 (*F* = 6.29, *p* = 0.003) and PHQ-15 (*F* = 4.95, *p* = 0.009) compared to the other two subgroups.

### 3.3 Correlations between various variables for the TMD group

Table 3 displays the correlation analyses of the variables from the TMD group. The oral behavior score was strongly associated with psychological outcomes, including somatization (*r* = 0.46), depression (*r* = 0.52) and anxiety (*r* = 0.52), and was moderately associated with chronic pain (*rs* = 0.23 ~ 0.28). There was only a near-moderate correlation with the masticatory function restriction score (*r* = 0.31), yet there was no correlation with motor function limitations, communication function limitations or the total JFL score. Additionally, psychological symptoms and pain were strongly correlated (*rs* = 0.31 ~ 0.60; Table 4).

**Table 3:** Comparisons of OBC, JFLS, psychological scale, chronic pain scale in TMD subgroup (*n* = 96).

Variables	Total (*n* = 96)		MPDS (*n* = 21)		Internal derangement (*n* = 55)		Osteoarthritis (*n* = 20)		*F*	*p*-value
	*M*	*SD*	*M*	*SD*	*M*	*SD*	*M*	*SD*		
OBC	20.96	6.10	23.14	4.36	20.71	5.76	19.35	7.96	2.14	0.124
JFLS	51.97	26.32	52.24	27.96	53.07	25.35	48.70	28.24	0.20	0.820
Masticatory	17.88	8.48	17.19	8.18	18.50	8.32	16.95	9.50	0.33	0.721
Motor	23.46	11.75	24.14	11.26	24.06	11.44	21.15	13.32	0.49	0.617
Communication	10.62	8.64	10.90	9.49	10.52	8.68	10.60	8.00	0.02	0.985
Psychological scale										
PHQ-9	9.93	4.03	12.48	2.50	9.27	3.74	9.05	5.05	5.97	**0.004**
GAD-7	8.16	3.27	10.19	2.60	7.80	3.08	7.00	3.58	6.29	**0.003**
PHQ-15	10.26	4.34	12.00	2.68	10.44	3.96	7.95	5.73	4.95	**0.009**
Chronic pain scale										
CPI	38.21	18.99	41.59	16.22	37.31	19.28	37.00	21.36	0.43	0.655
CPG	20.79	17.57	25.56	16.64	19.74	17.27	18.50	19.21	1.04	0.359
TDP	1.85	1.17	2.19	0.98	1.69	1.16	1.90	1.33	1.39	0.254

OBC, Oral Behavior Checklist; JFLS, Jaw functional limitation scale; CPI, Characteristic Pain Intensity; CPG, Chronic Pain Grade; TDP, Total Disability Points; MPDS, Myofascial pain and dysfunction syndrome group. Bold values indicate statistically significant results.

**Table 4:** Correlations between the various variables for patients with TMDs (*n* = 96).

No.	Variables	1	2	3	4	5	6	7	8	9	10	11
1	OBC	1										
2	JFLS_Ma	0.31**	1									
3	JFLS_Mo	0.18	0.84***	1								
4	JFLS_Co	0.04	0.62***	0.74***	1							
5	JFLS	0.19	0.90***	0.96***	0.86***	1						
6	PHQ-9	0.52***	0.34**	0.51***	0.15	0.39***	1					
7	GAD-7	0.52***	0.22*	0.28**	−0.03	0.19	0.87***	1				
8	PHQ-15	0.46***	0.46***	0.61***	0.30**	0.52***	0.83***	0.78***	1			
9	CPI	0.25*	0.73***	0.73***	0.33**	0.67***	0.60***	0.40***	0.60***	1		
10	CPG	0.28**	0.64***	0.72***	0.44***	0.67***	0.59***	0.39***	0.58***	0.87***	1	
11	TDP	0.23*	0.63***	0.74***	0.54***	0.71***	0.55***	0.31**	0.60***	0.81***	0.93***	1

*p < 0.05, **p < 0.01, ***p < 0.001. OBC, Oral Behavior Checklist; JFLS, Jaw functional limitation scale; JFLS_Ma, Masticatory function restriction score; JFLS_Mo, Motor function limitation score; JFLS_Co, Communication function limited score; PHQ-9, Patient Health Questionnaire-9; GAD-7, Generalized Anxiety Disorder Scale; PHQ-15, Patient Health Questionnaire-15; CPI, Characteristic Pain Intensity; CPG, Chronic Pain Grade; TDP, Total Disability Points.

### 3.4 Direct and indirect effects of pain on the relationships between oral behaviors and psychological outcomes

As shown in Models 1, 3, and 5 of Table 5, multiple regression analyses indicated that oral behavior could significantly predict depression (*β* = 0.56, *SE* = 0.04, *t* = 9.21, *p* < 0.001), anxiety (*β* = 0.56, *SE* = 0.03, *t* = 9.10, *p* < 0.001), and somatic symptoms (*β* = 0.51, *SE* = 0.04, *t* = 8.13, *p* < 0.001). As shown in Models 2, 4, and 6 of Table 5, regression analyses revealed that after controlling for covariates and oral behaviors, chronic pain still predicted psychological distress, including depression (*β* = 0.57, *SE* = 0.02, *t* = 7.05, *p* < 0.001), anxiety (*β* = 0.33, *SE* = 0.02, *t* = 3.39, *p* = 0.001), and somatic symptoms (*β* = 0.63, *SE* = 0.02, *t* = 8.37, *p* < 0.001; Table 6).

**Table 5:** Testing the mediation effects of pain on the relationships between oral behavior and psychological distress (*n* = 96).

Predictor variables	Model 1				Model 2				*R* ^2^	Adjusted *R*^2^
	*B*	*SE*	*t*	*p*	*B*	*SE*	*t*	*p*		
Depression										
Age	0.07	0.03	1.15	0.252	−0.25	0.03	−2.94	0.004	0.14**	0.12**
Marital Status	−0.16	0.50	−2.72	0.007	0.13	0.52	1.68	0.097		
Oral behavior	0.56	0.04	9.21	0.000	0.06	0.06	0.62	0.539	0.19*	0.16*
Pain					**0.57**	**0.02**	**7.05**	**0.000**	0.48***	0.46***
Anxiety										
	Model 3				Model 4					
Age	0.06	0.03	0.99	0.322	−0.17	0.03	−1.62	0.108	0.09*	0.07*
Marital Status	−0.16	0.43	−2.65	0.009	0.02	0.50	0.25	0.806		
Oral behavior	0.56	0.03	9.10	0.000	0.20	0.06	1.81	0.073	0.16**	0.13**
Pain					**0.33**	**0.02**	**3.39**	**0.001**	0.26**	0.22**
Somatic symptoms										
	Model 5				Model 6					
Age	0.05	0.03	0.78	0.434	−0.24	0.03	−3.02	0.003	0.13**	0.11**
Marital Status	−0.23	0.54	−3.74	0.000	0.10	0.53	1.31	0.195		
Oral behavior	0.51	0.04	8.13	0.000	0.05	0.06	0.58	0.567	0.18*	0.15*
Pain					**0.63**	**0.02**	**8.37**	**0.000**	0.54***	0.52***

*p < 0.05, **p < 0.01, ***p < 0.001. Each column is a regression model that predicts the criterion at the top of the column. Bold values indicate statistically significant results.

**Table 6:** Direct and indirect effects: the indirect effect of pain on the relation between oral behavior and mental health outcomes (*n* = 96).

Variables	Effect size	*SE*	*t*	*p*	95% Confidence interval
Depression					
Total effect	0.16	0.07	2.21	0.029*	0.017; 0.307*
Direct effect	0.04	0.06	0.62	0.539	−0.084; 0.160 (n.s.)
Indirect effect	0.12	0.05			0.028; 0.230^a^
Anxiety					
Total effect	0.16	0.06	2.75	0.007**	0.046; 0.284*
Direct effect	0.11	0.06	1.81	0.073	−0.010; 0.225 (n.s.)
Indirect effect	0.06	0.03			0.010; 0.124^a^
Somatic symptoms					
Total effect	0.19	0.08	2.33	0.022*	0.028; 0.344*
Direct effect	0.04	0.06	0.58	0.567	−0.089; 0.160 (n.s.)
Indirect effect	0.15	0.07			0.032; 0.288^a^

*p < 0.05; ns, not significantly.

a significantly indirect effect.

Bootstrapping mediation analyses further revealed significant indirect effects and nonsignificant direct effects, suggesting that oral behaviors had a significant effect on depression (effect size = 0.12, *SE* = 0.05, 95% CI = [0.028; 0.023]), anxiety (effect size = 0.06, *SE* = 0.03, 95% CI = [0.010; 0.124]), and somatic symptoms (effect size = 0.15, *SE* = 0.07, 95% CI = [0.032; 0.288]) completely through chronic pain.

## 4 Discussion

This cross-sectional, observational study assessed the psychological symptoms, including anxiety, depressive and somatic symptoms, of TMD patients and non-TMD individuals; examined the associations among oral behaviors, pain, jaw function and limitations, and psychological symptoms in TMD patients; and revealed the indirect effect of chronic pain on the relationships between oral behavior and psychological distress.

TMD patients exhibited significantly greater levels of psychological distress and dysfunctional oral behaviors than those without TMDs did, which was consistent with the findings of numerous studies on TMD symptoms (22, 24, 36). Many previous studies have demonstrated good reliability, validity, and clinical utility for the Axis II measures of depression, somatization, and graded chronic pain (46, 47). Another study suggested that anxiety, depression, and high pain catastrophizing are comorbid psychological conditions of TMDs (13). Therefore, it is necessary to analyze psychological symptoms for TMD diagnosis and treatment (6). Our analysis of psychological symptoms across TMD subgroups demonstrated that the MPDS subgroup exhibited significantly elevated levels of depression, anxiety, and somatic symptoms. As a muscle-origin pain disorder that often radiates beyond masticatory muscles, MPDS appears particularly associated with psychosocial factors (stress, emotional disturbances, depression/anxiety). Diffuse myofascial pain may cause heighten central nervous system arousal, which resulted in functional limitations, sleep disturbances, and poor mental health status (48). Previous studies reported similar results: the MPDS group presented more severe depressive and nonspecific physical symptoms (6, 35).

TMD patients tended to have a greater frequency of oral behaviors than non-TMD patients did, and their oral behaviors were positively associated with chronic pain and psychological outcomes, including depression, anxiety, and somatic symptoms. In line with a few previous studies examining the relationships between oral behaviors and psychological outcomes among TMD patients, these findings support the same results as those of the current study (15, 21, 49). Moreover, oral behaviors are positively associated with chronic pain (50), and chronic pain is closely linked to mental health outcomes among TMD patients, as demonstrated through many studies (34, 51). The fact that patients with painful TMD experience higher levels of depression and anxiety than those with nonpainful TMD also suggests this result (52). High pain catastrophizing was found to be associated with anxiety and depression for pain-related TMDs (13).

The mediation analysis in this study underscores pain has a significant indirect effect in the association between oral behaviors and psychological distress among TMD patients, a pattern indirectly supported by the findings of previous studies (33–35, 49). Pain appears to occupy a central position among DC/TMD Axis II indicators, demonstrating particularly robust associations with both psychological distress and oral behaviors (18), which was consistently observed in clinical practice. While the cross-sectional design necessitates cautious interpretation, converging evidence from multiple domains supports the predominant role of organic pain mechanisms in this relationship. This aligns well with established psychosomatic models wherein persistent parafunctional oral behavior initiates chronic pain that potentiate emotional distress (18, 36). Mindfulness-based interventions have demonstrated efficacy in chronic pain management (53, 54), which may be particularly relevant for TMD patients, as these approaches can improve individuals’ pain perception and acceptance, thereby enhancing pain coping. Our findings lend support to the conceptualization of psychological distress in TMD as frequently being secondary to chronic pain, suggesting that comprehensive management should prioritize addressing the pain.

This study has several limitations. First, as with all cross-sectional studies, our design cannot establish temporal relationships among oral behaviors, pain, and psychological distress. Prospective cohort studies are needed to elucidate potential causal pathways. Second, the moderate sample size may have limited the robustness of the SEM analyses, particularly in detecting complex mediation effects. And the smaller sample size in the MPDS subgroup might restrict certain analyses. Third, reliance on self-reported measures introduces potential recall bias, though this was mitigated through validated DC/TMD instruments. Future studies could incorporate both clinical examinations (e.g., Friction’s Craniomandibular Index) and advanced imaging (MRI/CT) to complement self-report data. Notwithstanding these limitations, our study provides a methodological framework for future mechanistic investigations while highlighting the clinical relevance of integrated pain-psychology assessment in TMD management.

The study underscores the clinical relevance of the TMD Axis II diagnostic criteria by providing empirical evidence for elevated psychological distress and dysfunctional oral behaviors in TMD patients, as well as their interrelationships. The findings offer preliminary insights into a potential mechanism, where chronic pain acts as a complete mediator, linking oral behaviors to psychological symptoms in this population. While the established associations between TMD, pain, and distress are recognized, this mediation model may help refine clinical understanding by suggesting that chronic pain could be a critical focus point for mitigating psychological distress. These observations may encourage further investigation into the underlying pathophysiology (e.g., neural sensitization) and prompt clinicians to consider pain management as part of a holistic diagnostic and therapeutic approach.

## 5 Conclusion

By emphasizing the Axis II diagnostic criteria, this study demonstrated the indirect effect of chronic pain on the associations between oral behaviors and psychological distress, including depression, anxiety and somatic symptoms, among TMD patients. Physicians should give more attention to patients’ chronic pain, which helps alleviate the psychological distress related to oral behaviors. This study contributes to the conceptual framework for the development of individualized diagnostic and therapeutic strategies for TMD patients.

## Data Availability

The raw data supporting the conclusions of this article will be made available by the authors, without undue reservation.
