# Supplementary material for: Correlations of oral behaviors, pain, and psychological distress among patients with temporomandibular disorders: clinical investigation of axial II evaluation
Source: Front Public Health. 2025 Jun 24;13:1604995. doi: 10.3389/fpubh.2025.1604995 (PMC12234292; doi:10.3389/fpubh.2025.1604995)
Supplement: Supplementary file 1 [file Data_Sheet_1.pdf]

Supplementary files

1. Graded Chronic Pain Scale – 1-month version
2. Jaw Functional Limitation Scale
3. The Oral Behavior Checklist
4. Generalized Anxiety Disorder 7 - Item Scale (GAD - 7)
5. Patient Health Questionnaire - 9 (PHQ - 9)
6. Patient Health Questionnaire - 15 (PHQ - 15)

Graded Chronic Pain Scale – 1-month version

1. How would you rate your facial pain **RIGHT NOW?** Use a scale from 0 to 10, where 0 is "no pain" and 10 is "pain as bad as could be".

No pain										Pain as bad as could be
0	1	2	3	4	5	6	7	8	9	10

2. In the <u>LAST 30 DAYS</u>, how would you rate your **WORST** facial pain? Use the same scale, where 0 is "no pain" and 10 is "pain as bad as could be".

No pain										Pain as bad as could be
0	1	2	3	4	5	6	7	8	9	10

3. In the <u>LAST 30 DAYS</u>, **ON AVERAGE**, how would you rate your facial pain? Use the same scale, where 0 is "no pain" and 10 is "pain as bad as could be". [That is, *your usual pain* at times you were in pain.]

No pain										Pain as bad as could be
0	1	2	3	4	5	6	7	8	9	10

4. In the <u>LAST 30 DAYS</u>, how many days did your facial pain keep you from doing your **USUAL ACTIVITIES** like work, school, or housework? (every day = 30 days)

______ Days

5. In the <u>LAST 30 DAYS</u>, how much has facial pain interfered with your **DAILY ACTIVITIES**? Use a 0-10 scale, where 0 is “no interference: and 10 is “unable to carry on any activities”.

No interference										Unable to carry on any activities
0	1	2	3	4	5	6	7	8	9	10

6. In the <u>LAST 30 DAYS</u>, how much has facial pain interfered with your **RECREATIONAL, SOCIAL AND FAMILY ACTIVITIES**? Use the same scale, where 0 is “no interference: and 10 is “unable to carry on any activities”.

No interference										Unable to carry on any activities
0	1	2	3	4	5	6	7	8	9	10

7. In the <u>LAST 30 DAYS</u>, how much has facial pain interfered with your **ABILITY TO WORK**, including housework? Use the same scale, where 0 is “no interference: and 10 is “unable to carry on any activities”.

No interference										Unable to carry on any activities
0	1	2	3	4	5	6	7	8	9	10

Copyright Von Korff M. Available at http://www.rdc-tmdinternational.org
Version 23Nov2020. No permission required to reproduce, translate, display, or distribute.

Scoring Guidelines for Graded Chronic Pain Scale – 1-month version[1]

Description

The 1-month Graded Chronic Pain Scale (GCPS) uses the same item structure as found in the original GCPS for 6 months, published in 1992.

Version 2.0 of the GCPS (6 months) was published in 2011. in addition to the 3 items for pain intensity and 4 items for function, one item was added to measure the number of days of pain. The additional item was not scored but served, instead, as an indicator for pain persistence. The DC/TMD includes a GCPS 1-month instrument that is structurally based on the GCPS 2.0 for 6 months and therefore includes the additional item measuring the number of pain days; adopting the GCPS 2.0 as the source instrument for the 1-month GCPS in the DC/TMD was made in recognition of the clinical usefulness of assessing pain persistence.

This version of the GCPS for measuring pain during the prior 30 days has been evaluated for reliability and validity, and only those items that were evaluated are included. This version therefore includes measurement only of pain intensity and pain-related disability during the past 30 days.

Scoring (item numbers refer to GCPS 1-month as attached to these scoring guidelines)

Characteristic Pain Intensity (CPI): compute mean of items 1-3 (pain right now, worst pain, average pain), and multiply by 10. Score range: 0-100.

Disability points for number of days with interference: assign points to item 4 based on below table.

Interference Score: compute mean of items 5-7 (daily activities, social activities, work activities), and multiply by 10. Score range: 0-100.

Disability points for the interference score: assign points based on the below table; the determination is the same for both time frames.

Points for Disability Days		Points for Interference Score	
Days	Points	Interference	Points
0-1	0	0-29	0
2	1	30-49	1
3-4	2	50-69	2
5-30	3	70-100	3

Total Disability Points = Points for Disability Days + Points for Interference Score.

[1] Adapted from: Ohrbach R and Knibbe W (2018) Diagnostic Criteria for Temporomandibular Disorders (DC/TMD): Scoring Manual for Self-Report Instruments. Available at www.rdc-tmdinternational.org.

Missing data

If more than one response is missing among items 1-3 (pain intensity) or among items 5-7 (function), the respective subscale should not be scored. Missing data for number of disability days precludes determination of chronic pain grade.

Interpretation

Determination of Chronic Pain Grade

Grade	Label	CPI	Total Disability Points
0	None	0	N/A
1	Low intensity pain, with none-low pain-related disability	< 50	< 3
2	High intensity pain, with none-low pain-related disability	≥ 50	< 3
	[2a] High intensity pain, without pain-related disability	≥ 50	0
	[2b] High intensity pain, with low pain-related disability	≥ 50	1-2
3	Moderately limiting	N/A	3 - 4
4	Severely limiting	N/A	5 - 6

References

Sharma S, Kallen MA, Ohrbach R (in review). Graded Chronic Pain Scale: Validation of 1-month reference frame. Submitted to *Journal of Pain*.

Von Korff M (2011). Assessment of chronic pain in epidemiological and health services research: Empirical bases and new directions. *Handbook of Pain Assessment*, Third Edition. DC Turk and R Melzack (Eds). New York, Guilford Press**:** 455-473.

Von Korff M, et al. (1992). Grading the severity of chronic pain. *Pain* **50**: 133-149.

Von Korff MR, et al. (1992). Research diagnostic criteria. Axis II: Pain-related disability and psychological status. In: SF Dworkin & L LeResche (Eds.), Research Diagnostic Criteria for Temporomandibular Disorders. *Journal of Craniomandibular Disorders, Facial and Oral Pain* **6**: 330-334.

Jaw Functional Limitation Scale

For each of the items below, please indicate the level of limitation **during the last month**. If the activity has been completely avoided because it is too difficult, then circle '10'. If you avoid an activity for reasons other than pain or difficulty, then leave the item blank.

		**No limitation**										**Severe Limitation**
1.	Chew tough food	0	1	2	3	4	5	6	7	8	9	10
2.	Chew hard bread	0	1	2	3	4	5	6	7	8	9	10
3.	Chew chicken (for example, prepared in oven)	0	1	2	3	4	5	6	7	8	9	10
4.	Chew crackers	0	1	2	3	4	5	6	7	8	9	10
5.	Chew soft food (for example, macaroni, canned or soft fruits, cooked vegetables, fish)	0	1	2	3	4	5	6	7	8	9	10
6.	Eat soft food requiring no chewing (for example, mashed potatoes, apple sauce, pudding, pureed food)	0	1	2	3	4	5	6	7	8	9	10
7.	Open wide enough to bite from a whole apple	0	1	2	3	4	5	6	7	8	9	10
8.	Open wide enough to bite into a sandwich	0	1	2	3	4	5	6	7	8	9	10
9.	Open wide enough to talk	0	1	2	3	4	5	6	7	8	9	10
10.	Open wide enough to drink from a cup	0	1	2	3	4	5	6	7	8	9	10
11.	Swallow	0	1	2	3	4	5	6	7	8	9	10
12.	Yawn	0	1	2	3	4	5	6	7	8	9	10
13.	Talk	0	1	2	3	4	5	6	7	8	9	10
14.	Sing	0	1	2	3	4	5	6	7	8	9	10
15.	Putting on a happy face	0	1	2	3	4	5	6	7	8	9	10
16.	Putting on an angry face	0	1	2	3	4	5	6	7	8	9	10
17.	Frown	0	1	2	3	4	5	6	7	8	9	10
18.	Kiss	0	1	2	3	4	5	6	7	8	9	10
19.	Smile	0	1	2	3	4	5	6	7	8	9	10
20.	Laugh	0	1	2	3	4	5	6	7	8	9	10

The Oral Behavior Checklist

How often do you do each of the following activities, based on **the last month**? If the frequency of the activity varies, choose the higher option. Please place a (√) response for each item and do not skip any items.

	**Activities During Sleep**	**None of the time**	**< 1 Night /Month**	**1-3 Nights /Month**	**1-3 Nights /Week**	**4-7 Nights/ Week**
1	Clench or grind teeth **when asleep**, based on any information you may have.	☐	☐	☐	☐	☐
2	Sleep in a position that puts pressure on the jaw (for example, on stomach, on the side).	☐	☐	☐	☐	☐
	**Activities During Waking Hours**	**None of the time**	**A little of the time**	**Some of the time**	**Most of the time**	**All of the time**
3	Grind teeth together **during waking hours.**	☐	☐	☐	☐	☐
4	Clench teeth together **during waking hours.**	☐	☐	☐	☐	☐
5	Press, touch, or hold teeth together other than while eating (that is, contact between upper and lower teeth).	☐	☐	☐	☐	☐
6	Hold, tighten, or tense muscles without clenching or bringing teeth together.	☐	☐	☐	☐	☐
7	Hold or jut jaw forward or to the side.	☐	☐	☐	☐	☐
8	Press tongue forcibly against teeth.	☐	☐	☐	☐	☐
9	Place tongue between teeth.	☐	☐	☐	☐	☐
10	Bite, chew, or play with your tongue, cheeks or lips.	☐	☐	☐	☐	☐
11	Hold jaw in rigid or tense position, such as to brace or protect the jaw.	☐	☐	☐	☐	☐
12	Hold between the teeth or bite objects such as hair, pipe, pencil, pens, fingers, fingernails, etc	☐	☐	☐	☐	☐
13	Use chewing gum.	☐	☐	☐	☐	☐
14	Play musical instrument that involves use of mouth or jaw (for example, woodwind, brass, string instruments).	☐	☐	☐	☐	☐
15	Lean with your hand on the jaw, such as cupping or resting the chin in the hand.	☐	☐	☐	☐	☐
16	Chew food on one side only.	☐	☐	☐	☐	☐
17	Eating between meals (that is, food that requires chewing).	☐	☐	☐	☐	☐
18	Sustained talking (for example, teaching, sales, customer service).	☐	☐	☐	☐	☐
19	Singing.	☐	☐	☐	☐	☐
20	Yawning.	☐	☐	☐	☐	☐
21	Hold telephone between your head and shoulders.	☐	☐	☐	☐	☐

Generalized Anxiety Disorder 7 - Item Scale (GAD - 7)

Please rate how often you have been bothered by the following problems in the past 2 weeks on a scale of 0 – 3 (0 = *Not at all*; 1 = *Several days*; 2 = *More than half the days*; 3 = *Nearly every day*):

		**Not at all**	**Several days**	**More than half the days**	**Nearly every day**
1	Feeling nervous, anxious, or on edge	0	1	2	3
2	Not being able to stop or control worrying	0	1	2	3
3	Worrying too much about different things	0	1	2	3
4	Trouble relaxing	0	1	2	3
5	Being so restless that it is hard to sit still	0	1	2	3
6	Becoming easily annoyed or irritable	0	1	2	3
7	Feeling afraid as if something awful might happen	0	1	2	3

Scoring:

- The total score is the sum of the scores of the 7 items, ranging from 0 to 21.
- 0 - 4: Minimal anxiety symptoms.
- 5 - 9: Mild anxiety.
- 10 - 14: Moderate anxiety.
- 15 - 21: Severe anxiety.

Patient Health Questionnaire - 9 (PHQ - 9)

Over the last 2 weeks, how often have you been bothered by the following problems (0 = *Not at all*; 1 = *Several days*; 2 = *More than half the days*; 3 = *Nearly every day*)?

		**Not at all**	**Several days**	**More than half the days**	**Nearly every day**
1	Little interest or pleasure in doing things	0	1	2	3
2	Feeling down, depressed, or hopeless	0	1	2	3
3	Trouble falling or staying asleep, or sleeping too much	0	1	2	3
4	Feeling tired or having little energy	0	1	2	3
5	Poor appetite or overeating	0	1	2	3
6	Feeling bad about yourself - or that you are a failure or have let yourself or your family down	0	1	2	3
7	Trouble concentrating on things, such as reading the newspaper or watching television	0	1	2	3
8	Moving or speaking so slowly that other people could have noticed? Or the opposite - being so fidgety or restless that you have been moving around a lot more than usual	0	1	2	3
9	Thoughts that you would be better off dead or of hurting yourself in some way	0	1	2	3

Scoring:

- The total score is the sum of the scores of the 9 items, ranging from 0 to 27.
- 0 - 4: Minimal depression symptoms.
- 5 - 9: Mild depression.
- 10 - 14: Moderate depression.
- 15 - 19: Moderately severe depression.
- 20 - 27: Severe depression.

Patient Health Questionnaire - 15 (PHQ - 15)

Please rate how much you have been bothered by each of the following problems in the past 4 weeks on a scale of 0 – 2 (0 = *Not bothered at all*; 1 = *Bothered a little*; 2 = *Bothered a lot*):

		**Not bothered at all**	**Bothered a little**	**Bothered a lot**
1	Stomach pain	0	1	2
2	Back pain	0	1	2
3	Headaches	0	1	2
4	Muscle aches	0	1	2
5	Joint pain	0	1	2
6	Chest pain	0	1	2
7	Heartburn or indigestion	0	1	2
8	Constipation, diarrhea, or loose stools	0	1	2
9	Feeling tired or having low energy	0	1	2
10	Trouble sleeping	0	1	2
11	Shortness of breath	0	1	2
12	Dizziness or light - headedness	0	1	2
13	Numbness or tingling in hands, feet, or other parts of the body	0	1	2
14	Skin problems (such as rashes, itching, or hives)	0	1	2
15	Menstrual problems (for women only)	0	1	2

Scoring:

- The total score is the sum of the scores of the 15 items, ranging from 0 to 30.
- 0 - 4: Minimal somatic symptoms.
- 5 - 9: Mild somatic symptoms.
- 10 - 14: Moderate somatic symptoms.
- 15 - 30: Severe somatic symptoms.
